# Utilization of the Healthy Eating Index in Cystic Fibrosis

**DOI:** 10.3390/nu14040834

**Published:** 2022-02-16

**Authors:** Rosara Milstein Bass, Alyssa Tindall, Saba Sheikh

**Affiliations:** 1 Division of Gastroenterology, Hepatology and Nutrition, The Children’s Hospital of Philadelphia, Philadelphia, PA 19104, USA; bassr@chop.edu (R.M.B.); tindalla@chop.edu (A.T.); 2 Department of Pediatrics, University of Pennsylvania, Philadelphia, PA 19104, USA; 3 Division of Pulmonary and Sleep Medicine, The Children’s Hospital of Philadelphia, Philadelphia, PA 19104, USA

**Keywords:** cystic fibrosis, nutrition, healthy eating index, dietary guidelines

## Abstract

(1) Background: Malnutrition has been a hallmark of cystic fibrosis (CF) for some time, and improved nutritional status is associated with improved outcomes. While individuals with CF historically required higher caloric intake than the general population, new CF therapies and improved health in this population suggest decreased metabolic demand and prevalence of overweight and obesity have increased. This study aimed to (a) examine diet quality in a population of young adults with CF using the Healthy Eating Index, a measure of diet quality in accordance with the U.S. Dietary Guidelines for Americans and (b) evaluate and describe how subcomponents of the HEI might apply to individuals with CF (2) Methods: 3-day dietary recalls from healthy adolescents and young adults with CF were obtained and scored based on the Healthy Eating Index (3) Results: Dietary recalls from 26 (14M/12F) adolescents and young adults with CF (ages 16–23), were obtained. Individuals with CF had significantly lower HEI scores than the general population and lower individual component scores for total vegetables, greens and beans, total fruits, whole fruits, total protein, seafood and plant protein and sodium (*p* values < 0.01 for all). (4) Conclusion: Dietary quality was poor in these healthy adolescents and young adults with CF. Given the increased prevalence of overweight and obesity in CF, updated dietary guidance is urgently needed for this population. The Healthy Eating Index may be a valuable tool for evaluating dietary quality in CF.

## 1. Introduction

Malnutrition has been a hallmark of cystic fibrosis since the disease was first described in 1938 [1]. While exocrine pancreatic insufficiency is the primary driver of malnutrition in individuals with CF, abnormalities of energy metabolism and gastrointestinal and hepatobiliary function also contribute [2,3]. The importance of improving nutritional status was first identified by Corey et al. in 1988, who identified that taller and heavier individuals with CF on unrestricted diet and appropriate pancreatic enzyme supplementation had improved survival compared to their peers, despite similar pulmonary function [4]. Subsequent analyses confirmed the relationship between z-scores for weight and BMI and longitudinal changes in forced expiratory volume in one second within 60% to 140% of predicted values (FEV_1_%) [5]. The clearly defined relationship between nutritional status and overall health has driven the most recent consensus recommendations for nutrition in people with CF, which endorse maintenance of normal ranges of weight and stature for age (BMI ≥ 50th percentile in children, ≥22 in adult women and ≥23 in adult men) through routine energy intake of 110–200% of standards for healthy population [6]. Meeting nutritional goals has historically been a challenge for individuals with CF. However, more recent studies of healthy youth and young adults with CF, have demonstrated consistent achievement of the recommended caloric recommendations often through a diet that is high in saturated fat and added sugar and low in fiber [7,8,9,10,11].

In fact, it is likely that some individuals with CF are consuming calories in excess of their metabolic needs, as overweight and obesity are now being reported in individuals with CF [12,13]. Overweight and obesity is expected to increase in prevalence in the CF population, as highly effective cystic fibrosis transmembrane regulator (CFTR) modulator use becomes widespread. Highly effective CFTR modulators have been associated with increased weight and BMI, and are now available for approximately 90% of individuals with CF [14,15,16,17]. The changing body composition of the CF population warrants additional investigation within the CF research community regarding (1) the impact of overweight and obesity on health in individuals with CF and (2) the need for better understanding as to how dietary quality can impact nutritional status and health outcomes in this population.

Additional guidance for nutrition in CF, designed to build upon the existing 2008 guidelines was provided by McDonald et al. and recommend an “age-appropriate, healthy diet, that emphasizes culturally appropriate foods associated with positive health outcomes in the general population, including vegetables, fruits, whole grains, seafood, eggs, beans, peas, nuts and seeds, dairy products, and meat and poultry” [18]. These specific food groups were not mentioned in the prior guidelines, and represent a shift in focus from diet quantity to diet quality. The Healthy Eating Index (HEI) incorporates all of these features and provides a metric for the assessment of dietary quality in the general population. The HEI is a measure of diet quality in accordance with U.S. Dietary Guidelines for Americans, independent of quantity [19]. While there is inconclusive and limited evidence regarding specific nutrients/food groups on outcomes in CF, it is likely that in aggregate, an overall healthy diet is beneficial for individuals with CF, and the HEI tool may provide a framework for dietary assessment and counseling in this population. This study aimed to (a) leverage the HEI for examination of diet quality in a population of young adults with CF and (b) evaluate and describe how subcomponents of the HEI scoring system might apply to individuals with CF.

## 2. Materials and Methods

### 2.1. Study Population

Individuals with CF from the CF Center at the Children’s Hospital of Philadelphia and the Hospital of the University of Pennsylvania were recruited for an ongoing longitudinal study examining body composition and muscle function in CF (ClinicalTrials.gov identifier: NCT02776098). Inclusion criteria for the trial were age 16–23 and confirmed diagnosis of CF. Exclusion criteria included chronic glucocorticoid use, organ transplantation, severe CF pulmonary disease (forced expiratory volume (FEV) 1%-predicted < 40%) and established diagnosis of CF related diabetes. While not part of recruitment criteria, the CF cohort was found to be was relatively healthy overall, as defined by median FEV_1_ 98% of predicted and median BMI 23.7 for males and 24.9 for females.

The dietary data for the general population were obtained from nationally representative survey data—the What We Eat In America (WWEIA) portion of the 2015–2016 National Health and Nutrition Examination Survey (NHANES) [20]. The WWEIA is the dietary intake interview component of the NHANES. In 2015–2016, 7918 Americans ages 2 and above (3877M/4041F) reported the types and quantities of foods and beverages they consume in a 24-h period through this interview. The Center for Nutrition Policy and Promotion calculated average HEI-2015 scores for the American population using this information [19].

### 2.2. Diet Recall

Three 24-h dietary recall interviews (2 weekdays and 1 weekend day) were collected by a Registered Dietitian Nutritionist via phone from each study participant. Interviews were conducted within the 3 week period following the study visit. The dates and times of the recalls were unannounced (unscheduled) so that participants did not change their normal eating pattern.

The 24-h dietary and supplement recall interviews elicited a detailed summary of all foods, beverages and dietary supplements consumed by participants during a complete 24-h period (from midnight-to-midnight) for the day preceding the interview. Information was obtained on the time of each eating occasion, the type of meal (breakfast, lunch, supper, snack), the location of the meal (home, school, other) and what and how much was consumed.

Data from these interviews were entered into Nutrition Data System for Research (NDSR) (Version NDS2021, MN, USA) for analysis.

### 2.3. Calculation of an HEI Score

HEI scores for the CF population were calculated using Stata 17.0. Dietary recall data were evaluated using percentage estimated energy requirement (%EER) and recall days were included if %EER was >30%. Subjects were included if at least two recall days met this criteria. NDSR output files 04 and 09 were used to calculate total HEI score and individual component scores according to the Healthy Eating Index-2015 scoring standards, which were also used in the NHANES population [21]. Mean scores were calculated for participants by taking the mean score of their HEI and component scores across their recall days.

### 2.4. Statistical Analysis

Stata 17.0 was used to analyze study data. Subjects were grouped by age according to the 2020–2025 Dietary Guidelines for Americans (14–18 and 19–30 years). Normality of the data was assessed using q–q plots [22]. The Kolmogorov–Smirnov test for nonparametric data was used to compare the mean total HEI score and mean component scores between the two age groups. One-sample t-tests were used to compare mean HEI scores and components with nationally representative mean HEI scores and component scores.

## 3. Results

### 3.1. Subject Characteristics

26 adolescents and young adults with CF (median age 19.8 (range: 16.5–23.2) and 14M/12F) were enrolled in the study. Of these subjects, 20 were pancreatic insufficient (PI) and 6 were pancreatic sufficient (PS). Subjects were generally healthy, as evidenced by median FEV_1_% predicted of 98% (71–129), and average BMI 23.7 (19.9–33.8) for males and 24.9 (20.2–28.4) for females (BMIs of >23 for males and >22 for females are associated with improved outcomes in CF). Five Subjects ages 16–18 had significantly lower weight (*p* = 0.009) and height (*p* = 0.04) than subjects ages 19–26, but better pulmonary function (*p* = 0.004), all likely associated with younger age (Table 1). Nine subjects (6M/3F) had BMI below CFF targets of 23 for males and 22 for females. Twelve subjects (6M/6F) had BMI > 25, which the WHO defines as overweight, and 1 subject (M) had BMI > 30, which the WHO defines as obese.

### 3.2. HEI Scores for CF vs. General Population

Individuals with CF had significantly lower scores than the general populations for total HEI score and within the following categories of the HEI (Table 2): total vegetables; greens and beans; total fruits; whole fruits; total protein; seafood and plant protein; and sodium. No difference in HEI metrics was seen between younger (age 16–18) and older (age 19–23) individuals with CF (Table 2) or when either of these age groups were compared to the US population (all *p* > 0.05, data not shown).

## 4. Discussion

This study examined dietary quality in a cohort of young, healthy adults with CF. Our data showed that younger individuals had poor dietary quality as measured by the Healthy Eating Index, and scores are lower than what have been reported for a healthy reference population. We found that although not statistically significant, 16–18 year olds tended to have worse diet quality than 19–23 year olds, which is consistent with trends seen in the general population. 

While multiple studies have examined dietary quality in CF, this study is unique as we leveraged the HEI, which specifically examines how dietary intake aligns with the recommendations for the Dietary Guidelines for Americans [19]. Alvarez et al. have also utilized this score to measure dietary quality in an older population (ages 18–50) of individuals with CF, and found individuals with CF had lower HEI scores as compared to age matched controls. The present study differs as our data were collected using a 3 day diet history obtained by a Registered Dietitian Nutritionist as opposed to a patient dietary records, we leveraged the NHANES database as a reference sample, and our CF population was healthier, as evidenced by their higher average FEV_1_ (98% vs. 74%) and higher average BMI (24.1 vs. 21.6). Although Alvarez et al. did not find a significant difference between CF and controls when examining specific elements of the HEI, we found that individuals with CF had lower scores than the general population in total HEI score and almost all HEI components. Specific dietary components have not been specifically linked with outcomes in CF; however, it is important to consider the deficits in the current CF diet, and how the benefits of each of the HEI components may specifically apply to individuals with CF.

### 4.1. Fruits, Vegetables and Legumes and Cystic Fibrosis

Our study found individuals with CF scored lower than average for consumption of vegetables, fruits and greens and beans. This is consistent with prior studies of intake in CF. In a cross-sectional study of 24 clinically stable adults with CF (mean FEV_1_% 60, BMI 22 kg/m^2^), none of the subjects met the recommended five servings of vegetables, and only 8.3% met daily fruit intake recommendations [23]. In an observational study by Calvo et al., which examined dietary patterns in 207 children with CF (ages 2–17) from six European CF centers, median intake of vegetable products was 1.0–2.8 times per day, fruits from 1.0–1.5 times per day and legumes from 0–0.2 times per day, all well below recommended daily intake [11].

Similar findings were seen in a Greek study of dietary quality in CF, only 16.7% of females (n = 44) and 17.1% of males (n = 32) consumed fruit more than once daily and only 14.6% of females and 20% of males consumed vegetables more than once daily. Additionally, this same study found that only 41.7% and 40% of females and males, respectively, consumed pulses on more than one occasion per week [7].

As fruits, vegetables and legumes are rich in fiber, not unexpectedly, overall daily fiber intake has been shown to be lower than recommended in individuals with CF, with median intakes ranging from 10.5–17.0 g/day in a study by Calvo et al. [11]. When Gavin et al. compared fiber intake in youth with CF to age-matched controls, mean daily fiber intake was lower in CF [24].

In addition to fruit, vegetable and legume fiber sources, whole grains are also an important source of dietary fiber. While individuals with CF still scored below target in this category, intake was not significantly lower than the general population, nor were intakes of refined grains or added sugar higher than the general population. No studies have specifically examined intake of whole grains as compared to refined grains in individuals with CF, but Sutherland et al. found significantly higher intake of refined carbohydrates including confectionary, packaged snacks, baked products and sweetened drinks in youth with CF as compared to age- and gender-matched controls.8 Additionally, Tierney et al. found in people with CF, 29% of daily caloric intake came from discretionary foods (packaged sweets, sugar sweetened beverages etc.), compared with 25% of non-CF adults.

The role of fiber in the CF diet has not been fully established. In a study of 68 children with CF, who were grouped based on presence of no, mild/moderate or severe abdominal pain, grams of fiber per kilogram body weight was significantly lower in the severe abdominal pain group than the others [24]. Contradictory results were seen by Proesman et al. who did not find a relationship between fiber intake and gastrointestinal problems in patients with CF [25].

Individuals with CF have been shown to have dyslipidemia, with elevated triglycerides and low HDL [26]. Dietary fiber has been shown to be inversely related to triglyceride levels in non-CF adults with overweight and obesity [27]. A cross-sectional study conducted in China found a dose-response relationship between increased dietary fiber intake and increase of HDL cholesterol in males [28]. Future studies should examine whether dietary fiber intake, similarly impacts lipid profiles of people with CF.

The quality of carbohydrate intake by individuals with CF, may also have implications for glycemic controls, as carbohydrates, total sugars, added sugars and dietary glucose load were found to be significantly positively associated with measures of glycemic variability in a cohort of adults with CF who were not on insulin or other glucose-lowering therapies [29]. 

### 4.2. Dairy and Cystic Fibrosis

Individuals with CF had suboptimal scores for dairy intake, but interestingly, scores were slightly higher than the general population. Currently, there are no specific guidelines for calcium intake in CF, and in the United States, it is recommended that people with CF adhere to the 1997 Institute of Medicine (IOM) guidelines for calcium intake for the general population [16,30]. Only 48.5% of Australian adults with CF met daily requirements for dairy intake [23]. In a study by Calvo et al., average calcium intake ranged from 614–1022 mg daily, below 1997 IOM recommendations for 1300–1500 mg daily [30]. Interestingly on food frequency questionnaires, dairy products were cited as the most frequently consumed food group among people with CF [11]. Vitamin D, which also plays an important role in bone health, is monitored annually, and as most individuals with CF are unable to meet needs through dietary intake alone, regardless of dairy consumption, dosing is based on serum levels, with adjustments as needed to achieve a target level of >30 ng/mL; however, there is a lack of international consensus on this goal [31,32].

With improved longevity in CF, CF bone disease (CFBD) is an emerging problem and can impact both pulmonary function and overall health [32]. As calcium and vitamin D play critical roles in bone formation and maintenance, it is important to better understand (a) the role that these specific nutrients play in the pathogenesis of CFBD and (b) the optimal intake that is necessary for individuals with CF, as this may be different than the general population.

### 4.3. Protein and Cystic Fibrosis

In our study, both total and seafood/plant protein were suboptimal in CF and lower than the reference population. Current recommendations are for 20% of calories in the CF diet come from protein [33]. Additionally, it has been shown that individuals with CF have lower lean muscle mass and increased systemic inflammation, which both are associated with increased dietary protein needs [34]. In a study by Calvo et al., protein intake was adequate in people with CF according to these recommendations, but in an earlier longitudinal study by Smith et al., intake was consistently less than the recommended 20% of total caloric intake [11,35]. Additionally, in patients with CF and exocrine pancreatic insufficiency (EPI), protein digestibility is severely impaired. While PERT can improve digestibility, the process remains severely delayed [36]. Thus, further studies are needed to examine (a) whether increased protein intake can improve lean body mass in people with CF and whether (b) protein digestion can be optimized in people with EPI.

Finally protein source deserves additional consideration. Even when protein intake is adequate in people with CF, our study echoed prior findings that these needs are primarily met through intake of meat and dairy products as opposed to plant based protein [11]. In a systematic review and meta-analysis of protein intake and all-cause mortality in the general population, intake of plant protein was associated with a lower risk of all cause and cardiovascular disease mortality. This inverse relationship remained significant in studies that controlled for energy, BMI and macronutrient intake [37]. It will be important to understand if these findings also apply in the CF population.

### 4.4. Saturated Fat and Cystic Fibrosis

A high fat diet has been a hallmark of nutritional therapy for CF, as fat malabsorption can occur even with optimal enzyme supplementation [33]. Historically, the recommendations for fat intake were not typically achieved in people with CF; however, in more recent years, these recommendations are consistently being met and exceeded. Our study found that individuals with CF did not consume significantly more total or saturated fat than the general population.

While a low fat diet has been associated with increased mortality in CF, a high fat diet without attention to type of dietary fat may also be problematic.4 Multiple studies have shown high saturated fat intake in individuals with CF [11,35]. Despite high total fat intake, individuals with CF are at risk of essential fatty acid deficiency. The composition of dietary fat intake has been shown to play a role in development of this condition, with intake of mono and polyunsaturated fat intake related to higher levels of essential fatty acids in CF [38].

Saturated fat intake has also been shown to play a role in the pathogenesis of cardiovascular disease. While individuals with CF were previously not to be thought to be at increased risk of cardiovascular disease, more recently, higher augmentation index, a composite vascular parameter of both arterial stiffness and global peripheral wave reflection, was shown to be higher in adults with CF [39]. These studies suggest that while a high fat diet likely remains important in CF, additional attention should be paid to the composition of dietary fat, with increased intake of mono and polyunsaturated fats, and decreased consumption of saturated fat.

### 4.5. Strengths, Limitations and Future Directions

This study is the first, to our knowledge, to examine the dietary quality of children and young adults using specific sub-scores of components of the HEI, which is pertinent to understanding dietary patterns during periods of growth and development in CF. Dietary data for this study were collected by Registered Dietitian Nutritionists using a 24-h recall method, not participant diet records. Additionally, all participants’ dietary data met the minimum requirements for inclusion based on %EER. Limitations include a cross-sectional study design; a small, geographically localized sample size; and no matched control group. Examining dietary data in a larger, more representative sample of people with CF would allow for greater generalizability. Additionally, while interviews regarding dietary intake are vulnerable to inaccurate reporting by subjects, the use of a trained diet technician in data collection allows for increased detail that is often missed when subjects report dietary intake independently.

## 5. Conclusions

In summary, a cohort of young, relatively healthy individuals with CF generally had poor dietary quality as measured by the Healthy Eating Index. In the context of increased prevalence of overweight and obesity, and improved longevity, additional research is warranted to determine the most appropriate dietary recommendations to support overall health in people with CF.

## Figures and Tables

**Table 1:** Subject Characteristics by Age: Younger individuals with Cystic Fibrosis (CF) had lower weight (*p* = 0.009) and height (*p* = 0.04), and higher forced expiratory volume in one second (FEV_1_)% predicted (*p* = 0.004) than older individuals with CF. Gender distribution, pancreatic status and BMI were not different between the two age groups (all *p* > 0.05).

	Total (n = 26)	Ages 16–18 (n = 9)	Ages 19–23 (n = 17)	*p*-Value
Sex	14M/12F	3M/6F	11M/6F	Pr = 0.127
Weight	64.9 [50.6; 107.5]	60.5 [50.6; 71.0]	69.8 [57.3; 107.5]	0.009
Height	166.7 [148.8; 185.1]	163.1 [151.4; 173.6]	171.8 [148.8; 185.1]	0.04
BMI	24.1 [19.9; 33.8]	22.1 [20.2; 26.8]	25.5 [19.9; 33.8]	0.07
Pancreatic Insufficiency	20Y/6N	6Y/3N	14Y/3N	Pr = 0.366
FEV_1_% Predicted	98 [71; 129]	109 [94; 120]	87 [71; 129]	0.004

**Table 2:** HEI scores CF vs. reference: Individuals with CF had significantly lower total HEI scores than the US population (*p* < 0.0001) and lower individual component scores for total vegetables, greens and beans, total fruits, whole fruits, total protein, seafood and plant protein and sodium (all *p* < 0.01). While younger (ages 16–18) individuals with CF tended to have lower total and component HEI scores than older (ages 19–26) individuals with CF, these differences were not statistically significant (all *p* > 0.05). * indicates clinical significance, *p* < 0.05.

			Mean ± SD Score			*p*-Values	
Variable	Possible Score	U.S. Population	CF Subjects			Within CF: 16–18 Y vs. 19–23 Y	U.S. vs. All CF Subjects
			All Subjects	16–18 Y (n = 7)	19–23 Y (n = 19)		
HEI total score	100	59	46.0 ± 12.1	45.0 ± 9.5	46.0 ± 13.2	0.61	<0.0001 *
Total vegetables	5	3.3	2.2 ± 1.1	1.8 ± 1.2	2.4 ± 1.1	0.34	0.0001 *
Greens and beans	5	3.1	0.8 ± 1.3	1.0 ± 1.0	0.7 ± 1.4	0.62	<0.0001 *
Total fruits	5	4.2	1.3 ± 1.4	1.6 ± 1.7	1.2 ± 1.3	1	<0.0001 *
Whole fruits	5	2.9	1.0 ± 1.3	0.9 ± 1.0	1.0 ± 1.4	1	<0.0001 *
Dairy	10	6	6.3 ± 3.1	6.7 ± 3.5	6.2 ± 3.0	0.62	0.58
Total protein	5	5	3.7 ± 1.0	3.6 ± 1.4	3.7 ± 0.8	0.68	<0.0001 *
Seafood and plant protein	5	5	1.5 ± 1.8	2.1 ± 2.1	1.3 ± 1.7	0.43	<0.0001 *
Whole grains	10	3	3.2 ± 2.7	2.9 ± 3.1	3.4 ± 2.6	0.43	0.65
Fatty acids	10	4.1	3.7 ± 2.6	3.4 ± 1.2	3.8 ± 3.0	0.72	0.4
Refined grains	10	6.4	5.5 ± 2.8	5.3 ± 3.0	5.6 ± 2.8	0.85	0.11
Sodium	10	3.7	5.0 ± 2.4	5.1 ± 2.5	5.0 ± 2.5	0.62	0.01 *
Added sugars	10	6.8	6.2 ± 3.0	6.5 ± 2.4	6.0 ± 3.2	0.66	0.28
Saturated fats	10	5.1	5.7 ± 2.8	4.6 ± 2.3	6.1 ± 2.9	0.18	0.28

## Data Availability

Data for CF participants are not publicly available. Dietary data for the general population were obtained from What We Eat In America (WWEIA) portion of the 2015–2016 National Health and Nutrition Examination Survey (NHANES).
